# Estimated glucose disposal rate predicts the risk of diabetic peripheral neuropathy in type 2 diabetes: A 5‐year follow‐up study

**DOI:** 10.1111/1753-0407.13482

**Published:** 2024-01-15

**Authors:** Yuanpin Zhang, Wanwan Sun, Qi Zhang, Yuetian Bai, Lijin Ji, Hangping Zheng, Xiaoming Zhu, Xiaoxia Liu, Shuo Zhang, Qian Xiong, Yiming Li, Lili Chen, Bin Lu

**Affiliations:** ^1^ Department of Endocrinology and Metabolism Huashan Hospital Fudan University Shanghai China; ^2^ Department of Endocrinology Shanghai Gonghui Hospital Shanghai China

**Keywords:** diabetic peripheral neuropathy, estimated glucose disposal rate, insulin resistance, Michigan neuropathy screening instrument, type 2 diabetes

## Abstract

**Background:**

Insulin resistance is associated with chronic complications of diabetes, including diabetic peripheral neuropathy (DPN). Estimated glucose disposal rate (eGDR), calculated by the common available clinical factors, was proved to be an excellent tool to measure insulin resistance in large patient population. Few studies have explored the association between eGDR and DPN longitudinally. Therefore, we performed the current study to analyze whether eGDR could predict the risk of DPN.

**Methods:**

In this prospective study, 366 type 2 diabetes (T2DM) subjects without DPN were enrolled from six communities in Shanghai in 2011–2014 and followed up until 2019–2020. Neuropathy was assessed by Michigan Neuropathy Screening Instrument (MSNI) at baseline and at the end of follow‐up.

**Findings:**

After 5.91 years, 198 of 366 participants progressed to DPN according to MNSI examination scores. The incidence of DPN in the low baseline eGDR (eGDR < 9.15) group was significantly higher than in the high baseline eGDR (eGDR ≥ 9.15) group (62.37% vs. 45.56%, *p* = .0013). The incidence of DPN was significantly higher in patients with sustained lower eGDR level (63.69%) compared with those with sustained higher eGDR level (35.80%). Subjects with low baseline eGDR (eGDR < 9.15) had significantly higher risk of DPN at the end of follow‐up (odds ratio = 1.75), even after adjusting for other known DPN risk factors.

**Conclusions:**

The 5‐year follow‐up study highlights the importance of insulin resistance represented by eGDR in the development of DPN in T2DM. Diabetic patients with low eGDR are more prone to DPN and, therefore, require more intensive screening and more attention.

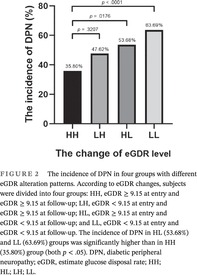

## Graphical abstract

- After 5.91 years, 198 of 366 (54.1%) participants with type 2 diabetes progressed to diabetic peripheral neuropathy (DPN).
- Subjects with low baseline estimated glucose disposal rate (eGDR < 9.15) had significantly higher risk of DPN at the end of follow‐up (odds ratio = 1.75), even after adjusting for other known DPN risk factors.
- Diabetic patients with low eGDR are more prone to DPN and, therefore, require more intensive screening and more attention.

## 1 INTRODUCTION

As the largest global epidemic of the 21st century, diabetes not only brings physical and psychological pains for the patients but also results in heavy social and economic burden.^1^ As the most common microvascular complication of diabetes, diabetic peripheral neuropathy (DPN) manifests with a so‐called stocking and glove distribution, severely affecting quality of life, especially in those with pain.^2^ The onset of DPN correlates with the duration of diabetes and up to 50% of patients develop DPN after 25 years of diabetes.^3^

Type 2 diabetes (T2DM) accounts for approximately 90% of all diabetic cases. Insulin resistance, the state of reduced responsiveness of target tissues to normal circulating insulin, is regarded as the central feature of T2DM and metabolic syndrome.^4^ Moreover, insulin resistance is associated with chronic complications of diabetes.^5^ The euglycemic hyperinsulinemic clamp technique is generally considered to be the gold standard for measuring insulin sensitivity in vivo.^6^ However, clamp technique is invasive and costly and thus not suitable for large‐scale clinical use. Estimated glucose disposal rate (eGDR), calculated by the readily available clinical factors including waist circumference (WC), hypertension (HT), and glycosylated hemoglobin (HbA1c), has been validated for the estimation of insulin resistance in individuals with type 1 diabetes (T1DM).^7^, ^8^, ^9^, ^10^, ^11^, ^12^ Recently, its potential to assess insulin resistance in T2DM has also been explored.^13^, ^14^, ^15^ However, few studies have explored the association between eGDR and DPN longitudinally, especially in the Chinese T2DM population. Therefore, we performed the current 5‐year follow‐up study to analyze whether eGDR could predict the risk of DPN in T2DM.

## 2 METHODS

### 2.1 Study design and participants

In this prospective study, type 2 diabetes subjects without DPN were enrolled from five communities in Jing'an District, Shanghai^16^ and one community in Baoshan District, Shanghai between 2011 and 2014, and followed up until 2019–2020. Inclusion criteria included (a) volunteered to participate in this study, (b) confirmed T2DM according to the American Diabetes Association diagnostic criteria,^17^ and (c) age ≥ 18 years. Exclusion criteria included (a) with previously diagnosed DPN, (b) peripheral neuropathy other than diabetic origin, (c) pregnancy, (d) lack of necessary clinical information, (e) major psychiatric disorders, and (f) acute infectious disease.

Written consent was obtained from all participants. The study was approved by the Institutional Review Broad of Huashan Hospital Fudan University.

### 2.2 Demographic and clinical data

The demographic data were collected from participants using a face‐to‐face structured interview by trained doctors and nurses, which included age, gender, family history of T2DM, and medications. Anthropometric measurements and blood pressure assessments were taken according to normative procedures. Body mass index (BMI) was calculated as weight divided by the square of height.

### 2.3 Laboratory examinations

A fasting venous blood sample was collected from all subjects. Fasting glucose were analyzed using the hexokinase method and an automatic analyzer (Hitachi 7600 chemical analyzer). The level of total cholesterol (TC), triacylglycerol (TG), high‐density lipoprotein cholesterol (HDL‐C), low‐density lipoprotein cholesterol (LDL‐C), serum creatinine (Scr), alanine aminotransferase (ALT), and aspartate aminotransferase (AST) were analyzed using an automatic analyzer (Hitachi 7600 chemical analyzer). Plasma HbA1c was determined by high pressure liquid chromatography using an analyzer (HLC‐723G7, Tosoh Corporation, Japan), with C‐peptide and insulin levels by chemiluminescence using an ADVIA Centaur XP automatic analyzer (Siemens Healthcare Diagnostics). The urinary albumin‐to creatinine ratio (ACR) was measured by Roche/Hitachi cobas c system (Roche Diagnostic GmbH, Mannheim, Germany).

### 2.4 Estimated glucose disposal rate

The eGDR (mg/kg/min) was calculated as previously described^8^: eGDR (mg × kg^−1^ × min^−1^) = 24.31−(12.22 × WHR)−(3.29 × HT)−(0.57 × HbA1c) [WHR = waist circumference/hip circumference, HT (yes = 1/no = 0), and HbA1c (%)]. HT was defined as treatment with antihypertensive medication or systolic blood pressure (SBP) ≥ 140 mm Hg, or diastolic blood‐pressure (DBP) ≥ 90 mm Hg, respectively.

The median of baseline eGDR level in this study was 9.15. Participants were divided into two groups based on the baseline eGDR level, low (eGDR < 9.15) and high baseline eGDR (eGDR ≥ 9.15) groups. After follow‐up, according to different eGDR alteration patterns, subjects were divided into four groups: HH, eGDR ≥ 9.15 at entry and eGDR ≥ 9.15 at follow‐up; LH, eGDR < 9.15 at entry and eGDR ≥ 9.15 at follow‐up; HL, eGDR ≥ 9.15 at entry and eGDR < 9.15 at follow‐up; and LL, eGDR < 9.15 at entry and eGDR < 9.15 at follow‐up.

### 2.5 Diagnosis of DPN

Michigan Neuropathy Screening Instrument (MNSI) is a well‐known instrument used to assess DPN with a sensitivity of 80% and a specificity of 95%.^18^, ^19^ As a validated, simple, noninvasive and inexpensive measurement tool, it is suitable for widely clinical application.

Symptoms and signs of lower limbs were recorded respectively. The assessments of the DPN were performed by one trained nurse using MNSI score: (a) foot deformities, dry skin, callus, infection; (b) foot ulceration; (c) semiquantitative assessment of vibration sensation at the dorsum of the great toe; and (d) grading of ankle reflexes. Responses were assigned scores of 1 (absent), 0.5 (decreased), or 0 (normal) for each foot. The total score of physical examination is 8 points and a score ≥2 is considered as DPN.^18^

### 2.6 DPN‐Check

DPN‐Check (Provided by Neurometrix Inc, Waltham, MA, USA), as a novel point‐of‐care nerve conduction device, was reported to provide an objective and noninvasive alternative method for the detection and diagnosis of DPN, with excellent correlations with standard nerve conduction study results.^20^, ^21^

The DPN‐Check was applied to evaluate DPN at the end of follow‐up. The device needs to be attached to a disposable biosensor, which facilitates electrical stimulation and integrates the nerve conduction data. The biosensor covered the area of the lateral aspect of the lower limb just above the ankle to reliably record sural nerve action potential responses. The whole process was carried out at room temperature and patients were evaluated bilaterally. The DPN‐Check test was considered abnormal when sural sensory nerve conduction amplitude was ≤4 μV and/or velocity was ≤40 m/s.

### 2.7 Statistical analysis

Normally distributed and continuous variables were expressed as means ± SDs and were analyzed by using Student *t* text. Categorical variables were presented as frequencies and proportions and analyzed by using nonparametric tests. Nonnormally distributed and continuous variables were expressed as median, 25th percentile, and 75th percentile (M[P25 ~ P75]).Spearman correlation analysis was used to analyze the relationship between baseline eGDR and relevant parameters at baseline or follow‐up. Multivariable logistic regression was used to evaluate the association between eGDR and DPN after adjusting for other variables. A *p* value <.05 was considered statistically significant. All statistical analyses were performed using SAS version 9.3 (SAS Institute Inc, Cary, NC, USA).

## 3 RESULTS

### 3.1 Baseline characteristics of the study participants grouped by the status of DPN at follow‐up

This study included 366 diabetic patients without DPN in 2011–2014. After 5.91 (4.67–9.46) years, 198 participants progressed to DPN according to MNSI examination scores, which were evaluated in 2019–2020 at follow‐up (Table 1).

**TABLE 1:** Characteristics of study participants grouped according to the absence or presence of DPN at follow‐up.

Variables	Non‐DPN	DPN	*p* value
	*n* = 168	*n* = 198	
Variables at baseline			
Baseline age (years)	61.30 ± 7.85	63.81 ± 8.37	.0036*
DM duration (years)	2.00 (0.00–9.00)	4.00 (0.00–12.00)	.1249
Male sex (*N*, %)	72, 42.86%	95, 47.98%	.3269
WC (cm)	86.20 ± 9.28	88.79 ± 10.45	.0133*
HC (cm)	95.90 ± 8.38	97.66 ± 8.32	.0452*
WHR	0.90 ± 0.06	0.91 ± 0.07	.1574
BMI (kg/m^2^)	24.57 ± 3.18	24.97 ± 3.61	.2711
HbA1c (%)	6.45 (5.80–7.50)	6.80 (5.90–8.00)	.0959
Fasting glucose (mmol/L)	7.55 ± 2.38	8.29 ± 2.99	.0106*
Fasting insulin (U/L)	8.01 (4.39–14.12)	8.35 (5.46–13.26)	.4122
C‐peptide (U/L)	1.47 (1.07–2.28)	1.58 (0.87–2.07)	.7896
SBP (mm Hg)	127.02 ± 16.28	130.25 ± 16.07	.0583
DBP (mm Hg)	78.42 ± 8.81	80.05 ± 8.67	.0775
HT (*N*, %)	9, 5.36%	23, 11.62%	.0346*
TG (mmol/L)	1.65 (1.18–2.16)	1.52 (1.11–2.22)	.8713
TC (mmol/L)	5.36 ± 0.97	5.28 ± 1.14	.5317
LDL‐C (mmol/L)	3.07 ± 0.71	2.98 ± 0.82	.3402
HDL‐C (mmol/L)	1.31 ± 0.28	1.34 ± 0.28	.3464
ALT (U/L)	24.00 (15.00–35.00)	28.00 (21.00–36.00)	.5937
AST (U/L)	23.00 (17.00–28.00)	24.50 (19.00–31.00)	.3568
Scr (μmol/L)	72.20 ± 18.43	71.69 ± 18.00	.8026
ACR (mg/g)	16.09 (8.05–55.47)	18.18 (8.84–42.20)	.6518
eGDR (mg × kg^−1^ × min^−1^)	9.22 ± 1.38	8.74 ± 1.71	.0043*
MNSI score	0.00 (0.00–1.00)	1.00 (0.00–1.00)	.6285
Use of insulin (*N*, %)	12, 7.14%	15, 7.58%	.8745
Use of metformin (*N*, %)	35, 20.83%	50,25.25%	.3184
Use of ACEI (*N*, %)	2, 1.19%	0	.2100
Use of ARB (*N*, %)	2, 1.19%	5, 2.53%	.6977
Use of CCB (*N*, %)	4, 2.38%	6, 3.03%	1.0000
Variables at follow‐up			
Follow‐up time	5.41 (4.36–9.46)	6.06 (4.79–9.42)	.2187
Follow‐up age (years)	67.42 ± 7.13	70.26 ± 7.72	.0003*
WC (cm)	86.05 ± 8.49	89.55 ± 9.29	.0002*
HC (cm)	96.14 ± 6.58	97.73 ± 9.91	.0787
WHR	0.89 ± 0.06	0.91 ± 0.06	.0092*
BMI (kg/m^2^)	24.43 ± 3.24	25.29 ± 4.02	.0260*
HbA1c (%)	6.70 (6.20–7.60)	7.00 (6.10–8.00)	.0468*
Fasting glucose (mmol/L)	7.08 ± 1.84	7.53 ± 2.86	.0811
Fasting insulin (U/L)	9.52 (5.45–15.35)	11.52 (7.63–23.18)	.0009*
C‐peptide (U/L)	0.71 (0.55–1.06)	0.77 (0.58–1.10)	.3700
SBP (mm Hg)	138.22 ± 18.29	142.29 ± 24.32	.0767
DBP (mm Hg)	77.63 ± 11.00	76.80 ± 10.83	.4721
HT (*N*, %)	79, 47.02%	118, 59.60%	.0162*
TG (mmol/L)	1.61 (1.15–2.21)	1.66 (1.11–2.20)	.9606
TC (mmol/L)	4.92 ± 1.16	5.17 ± 3.75	.4154
LDL‐C (mmol/L)	2.75 ± 0.94	2.73 ± 0.92	.8840
HDL‐C (mmol/L)	1.34 ± 0.36	1.36 ± 0.36	.5944
ALT (U/L)	17.50 (13.00–25.00)	19.00 (14.00–27.50)	.1863
AST (U/L)	19.00 (15.00–22.00)	19.00 (16.00–23.00)	.0539
Scr (mmol/L)	77.15 ± 24.33	78.56 ± 23.57	.5763
ACR (mg/g)	16.09 (8.05–55.47)	18.18 (8.84–42.20)	.8798
eGDR (mg × kg^−1^ × min^−1^)	7.81 ± 2.09	7.09 ± 2.17	.0010*
MNSI score	2.00 (2.00–2.00)	5.00 (4.00–6.00)	<.0001*
Left velocity (m/s)	55.00 (52.00–58.00)	54.00 (50.50–58.00)	.0419*
Right velocity (m/s)	55.00 (51.00–58.00)	52.00 (48.00–57.00)	.0359*
Left amplitude (μV)	13.00 (8.00–17.00)	11.00 (7.00–17.00)	.0348*
Right amplitude (μV)	12.00 (8.00–17.00)	10.00 (7.00–16.00)	.1694

*Note*: Significant *p* values are highlighted in bold and marked by*. Data are presented as means ± SDs, median (25th percentile–75th percentile), or *n* (percentage).

Abbreviations: ACEI, angiotensin‐converting enzyme inhibitor; ACR, urine microalbumin/urine creatinine ratio; ALT, alanine aminotransferase; ARB, angiotensin receptor blocker; AST, aspartate aminotransferase; BMI, body mass index; CCB, calcium channel blocker; DBP, diastolic blood pressure; DPN, diabetic peripheral neuropathy; eGDR, estimate glucose disposal rate; HbA1c, glycated hemoglobin; HC, hip circumference; HDL‐C, high‐density lipoprotein‐cholesterol; HT, hypertension; LDL‐C, low‐density lipoprotein‐cholesterol; MNSI, Michigan Neuropathy Screening Instrument; SBP, systolic blood pressure; Scr, serum creatinine; TC, total cholesterol; TG, triacylglycerol; WC, waist circumference; WHR, waist circumference/hip circumference.

The participants were divided into two groups based on the status of DPN at follow‐up. Compared with patients without DPN at follow‐up, those who developed DPN were older (63.81 ± 8.37 vs. 61.30 ± 7.85 years, *p* = .0036), had larger WC (88.79 ± 10.45 vs. 86.20 ± 9.28 cm, *p* = .0133), larger hip circumference (HC) (97.66 ± 8.32 vs. 95.90 ± 8.38 cm, *p* = .0452), higher level of fasting glucose (8.29 ± 2.99 vs. 7.55 ± 2.38 mmol/L, *p =* .0106), and lower level of eGDR (8.74 ± 1.71 vs. 9.22 ± 1.38 mg × kg^−1^ × min^−1^, *p* = .0043) at baseline. No significant differences were observed between the two groups in gender, diabetic duration, BMI, HbA1c, fasting insulin, C‐peptide, SBP, DBP, TG, TC, LDL‐C, HDL‐C, ALT, AST, Scr, ACR, MNSI score, and the use of metformin or angiotensin‐converting enzyme inhibitors (ACEI) at baseline (Table 1).

### 3.2 Follow‐up characteristics of the study participants grouped by the status of DPN

In the case of follow‐up, compared with patients without DPN, those with DPN were older (70.26 ± 7.72 vs. 67.42 ± 7.13 years, *p* = .0003), had larger WC (89.55 ± 9.29 vs. 86.05 ± 8.49 cm, *p* = .0002), higher BMI (25.29 ± 4.02 vs. 24.43 ± 3.24 kg/m,^2^ *p* = .0260), higher level of HbA1c (7.00 [6.10–8.00] vs. 6.70 [6.20–7.60] %, *p =* .0468), higher level of fasting insulin (11.52 [7.63–23.18] vs. 9.52 [5.45–15.35] U/L, *p =* .0009), lower level of eGDR (7.09 ± 2.17 vs. 7.81 ± 2.09 mg × kg^−1^ × min^−1^, *p* = .0010), and higher MNSI score (5.00 [4.00–6.00] vs. 2.00 [2.00–2.00], *p* < .0001). The DPN group had significantly lower left (54.00 [50.50–58.00] vs. 55.00 [52.00–58.00] m/s, *p* = .0419) and right nerve conduction velocity (52.00 [48.00–57.00] vs. 55.00 [51.00–58.00] m/s, *p* = .0359) and lower left nerve conduction amplitude (11.00 [7.00–17.00] μV vs. 13.00 [8.00–17.00] μV, *p* = .0348) measured by DPN‐Check. No significant differences were observed between the two groups in follow‐up time, HC, fasting glucose, C‐peptide, SBP, DBP, TG, TC, LDL‐C, HDL‐C, ALT, AST, Scr, and ACR level at follow‐up (Table 1).

### 3.3 The incidence of DPN was significantly higher in patients with low baseline eGDR

**FIGURE 1:**
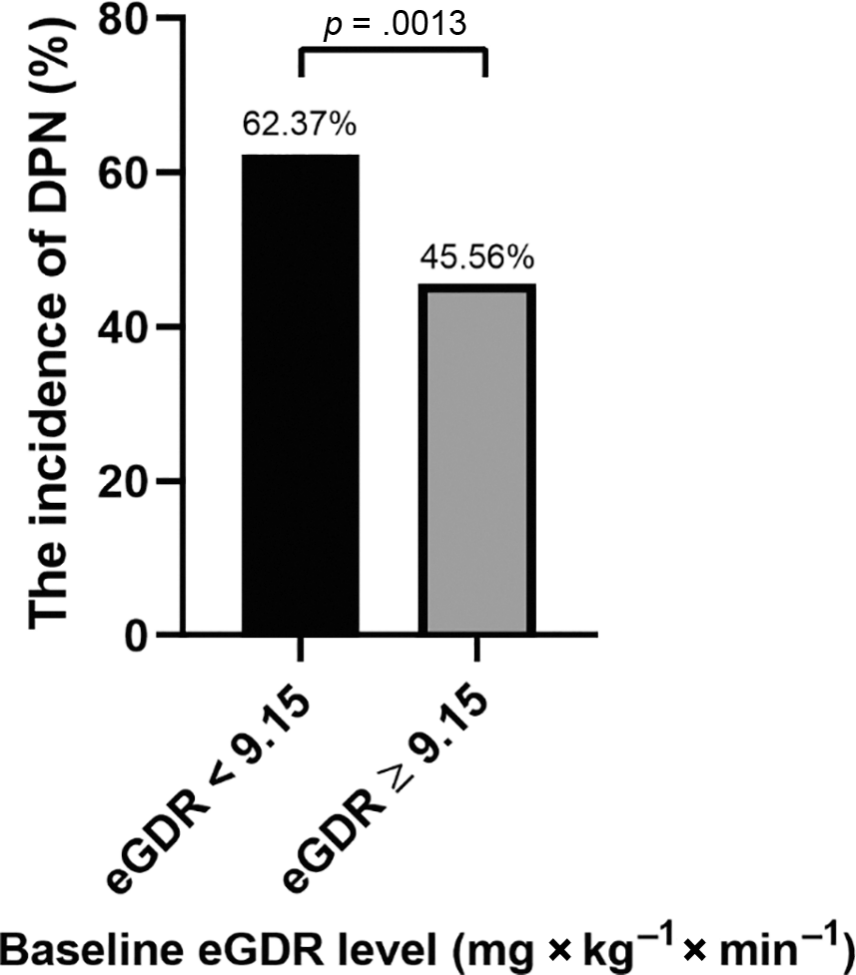
The incidence of DPN in low and high baseline eGDR groups. The incidence of DPN in low baseline eGDR (eGDR < 9.15) group is significantly higher than the high baseline eGDR (eGDR≥9.15) group (62.37% vs. 45.56%, *p* = .0013). DPN, diabetic peripheral neuropathy; eGDR, estimate glucose disposal rate.

As shown in Figure 1, at the end of follow‐up, the incidence of DPN in low baseline eGDR (eGDR < 9.15) group was significantly higher than in the high baseline eGDR (eGDR ≥ 9.15) group (62.37% vs. 45.56%, *p* = .0013).

### 3.4 The incidence of DPN was significantly higher in patients with sustained low eGDR level compared with those with sustained high eGDR level

**FIGURE 2:**
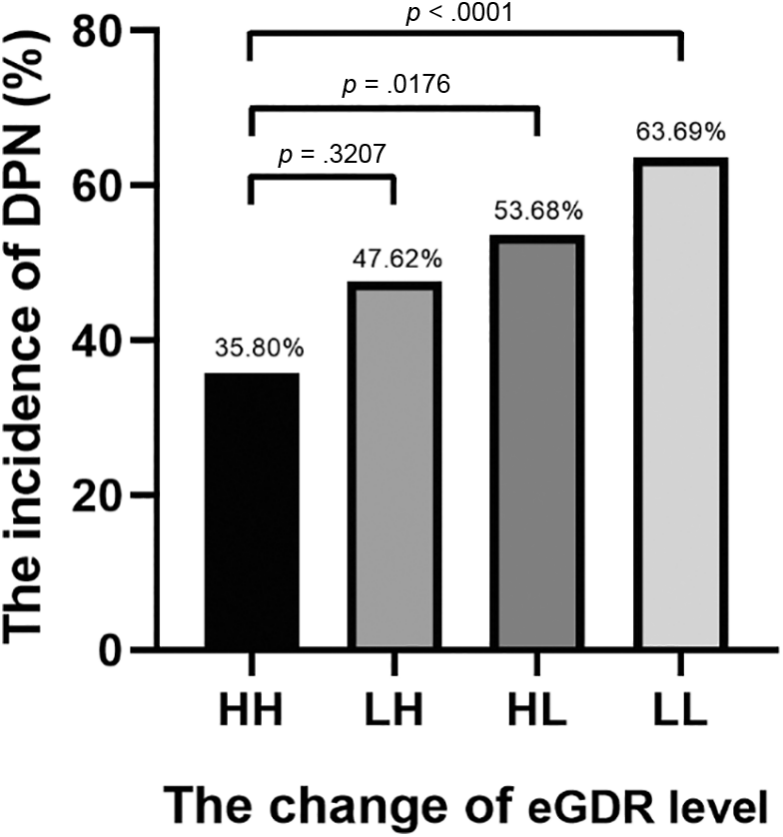
The incidence of DPN in four groups with different eGDR alteration patterns. According to eGDR changes, subjects were divided into four groups: HH, eGDR ≥ 9.15 at entry and eGDR ≥ 9.15 at follow‐up; LH, eGDR < 9.15 at entry and eGDR ≥ 9.15 at follow‐up; HL, eGDR ≥ 9.15 at entry and eGDR < 9.15 at follow‐up; and LL, eGDR < 9.15 at entry and eGDR < 9.15 at follow‐up. The incidence of DPN in HL (53.68%) and LL (63.69%) groups was significantly higher than in HH (35.80%) group (both *p* < .05). DPN, diabetic peripheral neuropathy; eGDR, estimate glucose disposal rate.

The participants were divided into four groups based on the changes of eGDR level: HH group, eGDR ≥ 9.15 at entry and eGDR ≥ 9.15 at follow‐up; LH group, eGDR < 9.15 at entry and eGDR ≥ 9.15 at follow‐up; HL group, eGDR ≥ 9.15 at entry and eGDR < 9.15 at follow‐up; and LL group, eGDR < 9.15 at entry and eGDR < 9.15 at follow‐up. The incidence of DPN in the HL (53.68%) and LL (63.69%) groups was significantly higher than in the HH (35.80%) group (both *p* < .05). (Figure 2).

**FIGURE 3:**
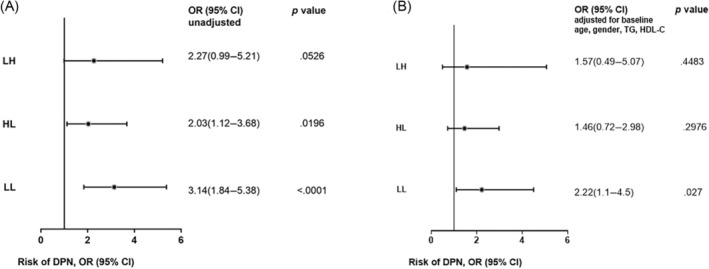
DPN risk in subjects with different eGDR alteration patterns. According to eGDR changes, subjects were divided into four groups: HH, eGDR ≥ 9.15 at entry and eGDR ≥ 9.15 at follow‐up; LH, eGDR < 9.15 at entry and eGDR ≥ 9.15 at follow‐up; HL, eGDR ≥ 9.15 at entry and eGDR < 9.15 at follow‐up; and LL, eGDR < 9.15 at entry and eGDR < 9.15 at follow‐up. (A) HL and LL groups had significantly higher risk of DPN compared to HH group (unadjusted). (B) After adjusting for age, gender, TG, HDL‐C, and use of metformin and ACEI at baseline, the LL group still had significantly higher risk of DPN compared to the HH group (OR = 2.22 [95% CI, 1.1–4.5]). Data are presented as odds ratios (95% confidence interval). ACEI, angiotensin‐converting enzyme inhibitor; CI, confidence interval; DPN, diabetic peripheral neuropathy; eGDR, estimate glucose disposal rate; HDL‐C, high‐density lipoprotein‐cholesterol; TG, triacylglycerol.

As shown in Figure 3, the HL and LL groups had significantly higher risk of DPN compared to the HH group (unadjusted). After adjusted for age, gender, TG, HDL‐C, and use of metformin and ACEI at baseline, the LL group still had significantly higher risk of DPN compared to HH group (odds ratio [OR] = 2.22 [95% confidence interval [CI], 1.1–4.5]).

### 3.5 Correlation of baseline eGDR with peripheral nerve conduction velocity and MNSI score at follow‐up

As shown in Table 2, Spearman correlation analysis revealed that baseline eGDR correlated negatively with baseline age (*r* = −0.20831, *p* < .0001) and baseline TG (*r* = −0.21867, *p* = .0002), and positively with baseline HDL‐C (*r* = 0.18193, *p* = .0021).

**TABLE 2:** Spearman correlation between baseline eGDR and variables at baseline or follow‐up.

Variables	Spearman correlation coefficient	*p* value
Baseline variables		
Baseline age (years)	−0.20831	<.0001*
Male gender	0.2483	<.0001*
MNSI score	−0.03080	.6603
TG (mmol/L)	−0.21867	**.0002**
TC (mmol/L)	−0.01598	.7872
LDL‐C (mmol/L)	−0.07147	.2291
HDL‐C (mmol/L)	0.18193	**.0021**
Follow‐up variables		
MNSI score	−0.16748	.002*
Left velocity (m/s)	0.2539	<.0001*
Right velocity (m/s)	0.2034	.0002*
Left amplitude (μV)	0.09546	.0792
Right amplitude (μV)	0.1072	.0546

*Note*: Significant *p* values are highlighted in bold and marked by*.

Abbreviations: eGDR, estimate glucose disposal rate; HDL‐C, high‐density lipoprotein‐cholesterol; LDL‐C, low‐density lipoprotein‐cholesterol; MNSI, Michigan Neuropathy Screening Instrument; TC, total cholesterol; TG, triacylglycerol.

Moreover, the baseline eGDR negatively correlated with follow‐up MNSI score (*r* = −0.16748, *p* = .0020) and positively correlated with left (*r* = 0.2539, *p* < .0001) and right (*r* = 0.2034, *p* = .0002) sural nerve conduction velocity by DPN‐Check performed at the end of follow‐up.

### 3.6 The prediction value of baseline eGDR on the risk of DPN

For a per‐1 unit decrease in baseline eGDR, the OR for DPN risk was 2.0 (95% CI, 1.32–3.03) by unconditional logistic regression. The multiple logistic regression model demonstrated that subjects with low baseline eGDR (eGDR < 9.15) had significantly higher risk of DPN in the end of follow‐up (OR, 1.75 [95% CI, 1.01–3.03]), even after adjusted for age, gender, HDL‐C, TG, and use of metformin and ACEI at baseline (Table 3).

**TABLE 3:** The prediction value of baseline eGDR on risk of DPN.

eGDR	OR (95% CI) Risk of DPN		
	Model 1	Model 2	Model 3
eGDR <9.15 at baseline	2.0 (1.32–3.03)	1.76 (1.03–3.01)	1.75 (1.01–3.03)
eGDR ≥9.15 at baseline	1	1	1

*Note*: Data are presented as odds ratios (95% confidence interval). Model 1: unadjusted. Model 2: adjusted for age, gender, HDL‐C, and TG at baseline. Model 3: adjusted for age, gender, HDL‐C, TG, and use of metformin and ACEI at baseline.

Abbreviations: ACEI, angiotensin‐converting enzyme inhibitors; CI, confidence interval; DPN, diabetic peripheral neuropathy; eGDR, estimate glucose disposal rate; HDL‐C, high‐density lipoprotein‐cholesterol; OR, odds ratio; TG, triacylglycerol.

## 4 DISCUSSION

DPN is a known strong predictor of diabetes‐related mortality, and it also acts as a significant independent risk factor for diabetic foot ulcers, the major cause of lower extremity amputation in diabetics.^22^ It was reported that the overall prevalence of DPN in the United Kingdom was 28.5%, which increased to 44% in patients over 70 years old.^23^ A national multicenter study revealed that the prevalence of DPN in China was 53%.^24^ The early manifestations of DPN are atypical and easy to be ignored, and DPN tends to enter an irreversible phase when diagnosed.^25^ Therefore, early and precise identification of patients at high risk of DPN is crucial for treatment.

Insulin is a neurotrophic factor, regulating the growth, survival, and differentiation of neurons.^26^ Insulin resistance is common in diabetic patients. Nevertheless, the direct effect of insulin resistance on the development of DPN still remains poorly understood.^4^ Like muscle and adipose tissue, neurons also could develop insulin resistance following hyperinsulinemia. Chronic insulin stimulation could result in the disruption of Akt signaling and mitochondrial biogenesis in neurons. The disruption of insulin signaling caused the loss of neurotrophic signal, inhibited the growth of nerve axons, and promoted cell apoptosis, which contributed to the development of DPN.^27^, ^28^ As the gold standard of insulin resistance, the euglycemic hyperinsulinemic clamp was not applicable for the large‐scale epidemiological studies because of its time cost, money cost, and invasiveness. Another commonly used parameter, homeostasis model assessment‐insulin resistance (HOMA‐IR), was usually calculated by blood glucose and insulin level. However, some studies showed that HOMA‐IR were not suitable for estimating insulin sensitivity in the unselected T2DM population such as the Renal Insufficiency and Cardiovascular Events (RIACE) cohort, because of the effect of exogenous insulin.^29^ The eGDR equation provided a potential tool for estimating insulin resistance using routinely collected clinical parameters. Zabala et al^14^ reported the good correlation between the euglycemic hyperinsulinemic clamp and eGDR in 24 male T2DM patients (*r* = 0.73). Penno et al^15^ further confirmed this correlation in 140 T2DM patients (*r* = 0.624). Moreover, its correlation with the euglycemic hyperinsulinemic clamp was better than that of HOMA‐IR in another validation study with 85 T1DM individuals.^29^

The eGDR has been used most frequently in studies of T1DM.^8^, ^11^, ^12^, ^30^, ^31^ The eGDR at baseline strongly predicted the development of retinopathy, nephropathy, and cardiovascular disease in the Diabetes Control and Complications Trial (DCCT).^12^ Furthermore, the decrease of eGDR has been associated with increased risk of diabetic complications/concomitant diseases including HT, peripheral artery disease, coronary artery calcification, retinopathy, albuminuria, peripheral neuropathy, and cardiac autonomic neuropathy in T1DM.^32^ Falkowski et al^11^ provided evidence of an association between eGDR and deteriorated olfactory function in adult T1DM patients. In addition, Uday et al used eGDR as a surrogate marker of insulin resistance in children with T1DM.^30^ Recently, eGDR has also been proposed as a method to estimate insulin resistance in T2DM patients. A nationwide population‐based cohort study was performed to investigate the association between eGDR and long‐term survival after coronary artery bypass grafting, suggesting that insulin resistance measured by eGDR could be a useful risk factor in patients with T2DM and ischemic heart disease.^13^ Zabala et al used the Swedish national diabetes register cohort to investigate the association between insulin resistance measured by eGDR and risk of first‐time stroke and mortality in T2DM population. They found that higher eGDR was associated with lower risk of stroke and death, independent of clinical characteristics and other identified risk factors for stroke and mortality.^14^ Gu et al also reported that decreased free thyroid hormone levels in normal range were associated with high glucose and insulin resistance, with FT3 and FT4 levels positively correlated with the eGDR in Chinese T2DM population.^33^

At present, there were still few studies on insulin resistance and the progression of diabetes neuropathy in T2DM population, especially lack of cohort study. A cross‐sectional study including 109 patients with T2DM revealed that insulin resistance, estimated by HOMA‐IR, is independently associated with diabetic cardiovascular autonomic neuropathy.^34^ It also reported that diabetic neuropathy can be affected by previous insulin resistance, estimated by the Kitt index, in a 6‐year follow‐up study including a total of 48 T2DM patients.^35^ However, few studies have explored the association between eGDR and DPN longitudinally. To our knowledge, the current study is the first prospective study to investigate the association of eGDR with DPN development in T2DM population. Our results showed the incidence of DPN in low baseline eGDR (eGDR < 9.15) group is significantly higher than the high baseline eGDR (eGDR ≥ 9.15) group (62.37% vs. 45.56%, *p* = .0013). The incidence of DPN was significantly higher in patients with sustained low eGDR level (63.69%, LL group) compared with those with sustained high eGDR level (35.80%, HH group). After adjusted for age, gender, TG, and HDL‐C, the LL group still had significantly higher risk of DPN compared to the HH group (OR = 2.22 [95% CI, 1.1–4.5]). Moreover, the baseline eGDR had a positive association with sural nerve conduction velocity at the end of follow‐up and correlated negatively with follow‐up MNSI score. Multiple logistic regression demonstrated that low baseline eGDR (eGDR < 9.15) was a risk factor for DPN (OR, 1.75 [95% CI, 1.01–3.03]), after adjusting for age, gender, HDL‐C, and TG at baseline. Thus, eGDR could help to predict the risk of DPN and provide reliable information for clinical decision making.

Limitations of our study included that the eGDR formula might not be as accurate as the euglycemic hyperinsulinemic clamp technique. However, eGDR equation is simpler and more suitable for large population‐based study. The eGDR is more practical in routine clinical practice. It was also reported that eGDR correlated significantly with clamp‐derived data from T2DM individuals, better than HOMA‐IR.^15^ A recent study revealed that the cutoff value of eGDR that reflects insulin resistance was 6.4 in T1DM patients in Lithuania.^10^ There is still a lack of research on the cutoff value of eGDR in T2DM, especially in the Chinese population, which needs further study. The participants in our study were enrolled from the natural population in six communities in Shanghai, China. We further analyzed the level of eGDR in people with or without metabolic syndrome and found that the mean value of those with metabolic syndrome was 8.57 and those without was 9.60. Thus, it is reasonable to group subjects by 9.15, which is the median of baseline eGDR level.

Moreover, our analysis was confined to the aged in a Chinese community cohort and it is unclear whether our results could apply to other populations. Another limitation is the relatively small sample size and the conclusion should be confirmed in larger sample studies.

## 5 CONCLUSIONS

The current 5‐year follow‐up study highlights the importance of insulin resistance represented by eGDR in the development of DPN in T2DM. Diabetic patients with low eGDR are more prone to DPN and, therefore, require more intensive screening and more attention.

## AUTHOR CONTRIBUTIONS

Yuanpin Zhang and Wanwan Sun wrote the manuscript. Yuanpin Zhang, Wanwan Sun, Qi Zhang, Yuetian Bai, Lijin Ji, Hangping Zheng, Shuo Zhang, and Xiaoxia Liu collected the data. Xiaoming Zhu, Shuo Zhang, and Wanwan Sun analyzed the data. Lili Chen, Bin Lu, and Yiming Li revised the manuscript. Yiming Li, Lili Chen, and Bin Lu conducted the study design and quality control. All authors read and approved the final manuscript.

## FUNDING INFORMATION

The present study was supported by grants from the National Natural Science Foundation of China (81770807, to B Lu and 81800692, to LJ Ji), Shanghai Talent Development Fund Program (2018054, to B Lu), Shanghai Science and Technology Committee Program (17411961500, to S Zhang), Shanghai General Hospital Program of Chinese traditional and Western medicine combination (ZY (2018‐2020)‐FWTX‐1002, to YM Li), Ministry of Science and Technology Program (2017ZX09304005, to S Zhang), the initial funding of Huashan Hospital (2021QD023, to Wanwan Sun), and Shanghai Municipal Commission of Health and Family Planning Clinical Research Project (20184Y0318, to LJ Ji).

## CONFLICT OF INTEREST STATEMENT

The authors declare that there is no conflict of interest.

## References

[1] Tabish SA . Is diabetes becoming the biggest epidemic of the twenty‐first century? Int J Health Sci. 2007;1(2):V‐VIII. PMC3068646 21475425

[2] Feldman EL , Callaghan BC , Pop‐Busui R , et al. Diabetic neuropathy. Nat Rev Dis Primers. 2019;5(1):41. 31197153 10.1038/s41572-019-0092-1

[3] Boulton AJ , Vinik AI , Arezzo JC , et al. Diabetic neuropathies: a statement by the American Diabetes Association. Diabetes Care. 2005;28(4):956‐962. 15793206 10.2337/diacare.28.4.956

[4] Kim B , Feldman EL . Insulin resistance in the nervous system. Trends Endocrinol Metab. 2012;23(3):133‐141. 22245457 10.1016/j.tem.2011.12.004 PMC3392648

[5] Han L , Ji L , Chang J , et al. Peripheral neuropathy is associated with insulin resistance independent of metabolic syndrome. Diabetol Metab Syndr. 2015;7:14. 25774226 10.1186/s13098-015-0010-y PMC4359792

[6] DeFronzo RA , Tobin JD , Andres R . Glucose clamp technique: a method for quantifying insulin secretion and resistance. Am J Physiol. 1979;237(3):E214‐E223. 382871 10.1152/ajpendo.1979.237.3.E214

[7] Epstein EJ , Osman JL , Cohen HW , Rajpathak SN , Lewis O , Crandall JP . Use of the estimated glucose disposal rate as a measure of insulin resistance in an urban multiethnic population with type 1 diabetes. Diabetes Care. 2013;36(8):2280‐2285. 23596179 10.2337/dc12-1693 PMC3714518

[8] Chillaron JJ , Goday A , Flores‐Le‐Roux JA , et al. Estimated glucose disposal rate in assessment of the metabolic syndrome and microvascular complications in patients with type 1 diabetes. J Clin Endocrinol Metab. 2009;94(9):3530‐3534. 19584183 10.1210/jc.2009-0960

[9] Pop A , Clenciu D , Anghel M , et al. Insulin resistance is associated with all chronic complications in type 1 diabetes. J Diabetes. 2016;8(2):220‐228. 25753338 10.1111/1753-0407.12283

[10] Simoniene D , Platukiene A , Prakapiene E , Radzeviciene L , Velickiene D . Insulin resistance in type 1 diabetes mellitus and its association with patient's micro‐ and macrovascular complications, sex hormones, and other clinical data. Diabetes Ther. 2020;11(1):161‐174. 31792784 10.1007/s13300-019-00729-5 PMC6965600

[11] Falkowski B , Duda‐Sobczak A , Araszkiewicz A , et al. Insulin resistance is associated with impaired olfactory function in adult patients with type 1 diabetes: a cross‐sectional study. Diabetes Metab Res Rev. 2020;36(6):e3307. 32129918 10.1002/dmrr.3307

[12] Kilpatrick ES , Rigby AS , Atkin SL . Insulin resistance, the metabolic syndrome, and complication risk in type 1 diabetes: “double diabetes” in the diabetes control and complications trial. Diabetes Care. 2007;30(3):707‐712. 17327345 10.2337/dc06-1982

[13] Nystrom T , Holzmann MJ , Eliasson B , Svensson AM , Kuhl J , Sartipy U . Estimated glucose disposal rate and long‐term survival in type 2 diabetes after coronary artery bypass grafting. Heart Vessels. 2017;32(3):269‐278. 27401741 10.1007/s00380-016-0875-1

[14] Zabala A , Darsalia V , Lind M , et al. Estimated glucose disposal rate and risk of stroke and mortality in type 2 diabetes: a nationwide cohort study. Cardiovasc Diabetol. 2021;20(1):202. 34615525 10.1186/s12933-021-01394-4 PMC8495918

[15] Penno G , Solini A , Orsi E , et al. Insulin resistance, diabetic kidney disease, and all‐cause mortality in individuals with type 2 diabetes: a prospective cohort study. BMC Med. 2021;19(1):66. 33715620 10.1186/s12916-021-01936-3 PMC7962330

[16] Zheng H , Sun W , Zhang Q , et al. Proinflammatory cytokines predict the incidence of diabetic peripheral neuropathy over 5 years in Chinese type 2 diabetes patients: a prospective cohort study. EClinicalMedicine. 2021;31:100649. 33385123 10.1016/j.eclinm.2020.100649 PMC7772538

[17] American Diabetes Association Professional Practice C . 2. Classification and diagnosis of diabetes: standards of medical care in diabetes‐2022. Diabetes Care. 2022;45(Suppl 1):S17‐S38. 34964875 10.2337/dc22-S002

[18] Herman WH , Pop‐Busui R , Braffett BH , et al. Use of the Michigan neuropathy screening instrument as a measure of distal symmetrical peripheral neuropathy in type 1 diabetes: results from the diabetes control and complications trial/epidemiology of diabetes interventions and complications. Diabet Med. 2012;29(7):937‐944. 22417277 10.1111/j.1464-5491.2012.03644.x PMC3641573

[19] Moghtaderi A , Bakhshipour A , Rashidi H . Validation of Michigan neuropathy screening instrument for diabetic peripheral neuropathy. Clin Neurol Neurosurg. 2006;108(5):477‐481. 16150538 10.1016/j.clineuro.2005.08.003

[20] Perkins BA , Grewal J , Ng E , Ngo M , Bril V . Validation of a novel point‐of‐care nerve conduction device for the detection of diabetic sensorimotor polyneuropathy. Diabetes Care. 2006;29(9):2023‐2027. 16936147 10.2337/dc08-0500

[21] Shibata Y , Himeno T , Kamiya T , et al. Validity and reliability of a point‐of‐care nerve conduction device in diabetes patients. J Diabetes Investig. 2019;10(5):1291‐1298. 10.1111/jdi.13007 PMC6717804 30659760

[22] Boulton AJ , Vileikyte L , Ragnarson‐Tennvall G , Apelqvist J . The global burden of diabetic foot disease. Lancet. 2005;366(9498):1719‐1724. 16291066 10.1016/S0140-6736(05)67698-2

[23] Young MJ , Boulton AJ , MacLeod AF , Williams DR , Sonksen PH . A multicentre study of the prevalence of diabetic peripheral neuropathy in the United Kingdom hospital clinic population. Diabetologia. 1993;36(2):150‐154. 8458529 10.1007/BF00400697

[24] Zhao Z , Ji L , Zheng L , et al. Effectiveness of clinical alternatives to nerve conduction studies for screening for diabetic distal symmetrical polyneuropathy: a multi‐center study. Diabetes Res Clin Pract. 2016;115:150‐156. 27116903 10.1016/j.diabres.2016.01.002

[25] Selvarajah D , Kar D , Khunti K , et al. Diabetic peripheral neuropathy: advances in diagnosis and strategies for screening and early intervention. Lancet Diabetes Endocrinol. 2019;7(12):938‐948. 31624024 10.1016/S2213-8587(19)30081-6

[26] Toth C , Brussee V , Martinez JA , McDonald D , Cunningham FA , Zochodne DW . Rescue and regeneration of injured peripheral nerve axons by intrathecal insulin. Neuroscience. 2006;139(2):429‐449. 16529870 10.1016/j.neuroscience.2005.11.065

[27] Kim B , McLean LL , Philip SS , Feldman EL . Hyperinsulinemia induces insulin resistance in dorsal root ganglion neurons. Endocrinology. 2011;152(10):3638‐3647. 21810948 10.1210/en.2011-0029 PMC3176655

[28] Kim B , Sullivan KA , Backus C , Feldman EL . Cortical neurons develop insulin resistance and blunted Akt signaling: a potential mechanism contributing to enhanced ischemic injury in diabetes. Antioxid Redox Signal. 2011;14(10):1829‐1839. 21194385 10.1089/ars.2010.3816 PMC3078499

[29] Williams KV , Erbey JR , Becker D , Arslanian S , Orchard TJ . Can clinical factors estimate insulin resistance in type 1 diabetes? Diabetes. 2000;49(4):626‐632. 10871201 10.2337/diabetes.49.4.626

[30] Uday S , Gorman S , Feltbower RG , Mathai M . Ethnic variation in the correlation between waist to height ratio and total daily insulin requirement in children with type 1 diabetes: a cross‐sectional study. Pediatr Diabetes. 2017;18(2):128‐135. 26843216 10.1111/pedi.12363

[31] Danielson KK , Drum ML , Estrada CL , Lipton RB . Racial and ethnic differences in an estimated measure of insulin resistance among individuals with type 1 diabetes. Diabetes Care. 2010;33(3):614‐619. 20007942 10.2337/dc09-1220 PMC2827519

[32] Mao Y , Zhong W . Changes of insulin resistance status and development of complications in type 1 diabetes mellitus: analysis of DCCT/EDIC study. Diabetes Res Clin Pract. 2022;184:109211. 35066056 10.1016/j.diabres.2022.109211

[33] Gu L , Yang J , Gong Y , et al. Lower free thyroid hormone levels are associated with high blood glucose and insulin resistance; these normalize with metabolic improvement of type 2 diabetes. J Diabetes. 2021;13(4):318‐329. 32981234 10.1111/1753-0407.13118

[34] Liu Y , Peng Y , Jin J , et al. Insulin resistance is independently associated with cardiovascular autonomic neuropathy in type 2 diabetes. J Diabetes Investig. 2021;12(9):1651‐1662. 10.1111/jdi.13507 PMC8409868 33460512

[35] Cho YN , Lee KO , Jeong J , et al. The role of insulin resistance in diabetic neuropathy in Koreans with type 2 diabetes mellitus: a 6‐year follow‐up study. Yonsei Med J. 2014;55(3):700‐708. 24719137 10.3349/ymj.2014.55.3.700 PMC3990070

